# Intracellular monitoring by dendritic cells – a new way to stay informed – from a simple scavenger to an active gatherer

**DOI:** 10.3389/fimmu.2022.1053582

**Published:** 2022-10-27

**Authors:** Christopher Herbst, Larry A. Harshyne, Botond Z. Igyártó

**Affiliations:** ^1^ Department of Microbiology and Immunology, Thomas Jefferson University, Philadelphia, PA, United States; ^2^ Department of Medical Oncology, Thomas Jefferson University, Philadelphia, PA, United States

**Keywords:** dendritic cells, scavenging, intracellular monitoring, surveillance, antigen uptake/presentation

## Abstract

Dendritic cells (DCs) are required for the initiation of the adaptive immune response. Their ability to acquire antigens in the periphery is a critical step in this process. DCs express a wide variety of adhesion molecules and possess an extremely fluid plasma membrane that facilitates scavenging the extracellular environment and capturing material like exosomes, apoptotic bodies, and pathogens. Besides these standard routes, the acquisition of antigens by DCs can be further facilitated by tunneling nanotubes, trogocytosis, and gap junctions. However, in this article, we will argue that this is an incomplete picture, as certain observations in the literature cannot be explained if we assume DCs acquire antigens only through these means. Instead, it is more likely that DCs preferentially use adhesion molecules to form long-lasting cell-cell interactions to actively siphon material from cells they are in direct contact with. It is highly likely that DCs use this mechanism to continually capture membrane and cytosolic material directly from surrounding cells, which they scan to assess the health of the donor cell. Doing so would provide an array of advantages for the host immune system, as it would not be reliant on compromised cells to release antigens into the extracellular milieu. Therefore, we propose updating our view of DC antigen acquisition to include a process of active, contact-dependent capture of material directly from neighboring cell cytosol (cytocytosis), which we would term *intracellular monitoring*.

## Standard routes that could contribute to antigen acquisition and immune monitoring

Cells acquire material from their surroundings *via* phagocytosis, endocytosis, and macropinocytosis. As the sentinels of the immune system, dendritic cells conduct these processes at an especially high rate to scan for potential threats. While the assorted means of antigen uptake vary widely in their mechanisms, all involve picking up extracellular material that has been released by other cells (1–3). This is problematic in the context of infectious disease or cancer, as selective pressure will drive pathogens or cancer cells to prevent the release of antigen. Many such mechanisms have been reported for intracellular pathogens (4–6). In response, dendritic cells must have evolved means of collecting antigen directly from surrounding cells that do not rely on compromised cells releasing antigen into the extracellular environment. What mechanisms have been described so far that could account for antigen acquisition?

## Tunneling nanotubes

Tunneling nanotubes (TNTs) are a mode of cell-cell communication that enable open ended connection between cells that are sessile and localized at a distance (7). TNTs transfer a wide variety of cellular material such as vesicles (8, 9), mitochondria (10), miRNAs (11), viral particles (12), proteins (13, 14), and mRNAs (15). The material transfer between the cells through TNTs is mainly mediated by actin and actin-binding motor proteins (7, 8). DCs have been reported to connect with each other using TNTs (16), which they can use to alert one another to the presence of bacterial supernatant *via* calcium signaling (17), so it is possible that the TNTs might play a role in the monitoring of their neighbors. However, its dependency on cytoskeletal connections between the cells and involvement of motor proteins for transport across the TNTs suggest selectivity in the material that can be exchanged, and thus limiting its use in immune surveillance.

## Gap junctions

Gap junctions are aggregates of intercellular channels that permit direct cell–cell transfer of ions and small molecules <1KDa (18). It is also well established that antigenic peptides can transfer through them (19). Thus, the role of gap junctions is likely limited in material transfer due to strict limits on the size of transferred molecules, nevertheless, their possible contribution to monitoring and antigen acquisition should not be ignored. Furthermore, the gap junction interface may represent a synapse where vesicles pass from one cell to another. Migratory DCs entering a tumor draining lymph node are known to transfer tumor antigen to other DCs in a synapse like process (20). While intriguing, this type of transfer would require cooperation of both cells, making it susceptible to interference and less likely to play a dominant role in monitoring.

## Trogocytosis

Trogocytosis is the process by which one cell “rips off” small portions of the target cell membrane without killing it (21–23). Trogocytosis is also routinely used by DCs (24, 25). While most groups studying this process have focused on the acquisition of membrane bound peptide-MHC for the purpose of T cell stimulation, it is possible that DCs also take the opportunity to collect and scan cytosolic material. Trogocytosis likely does not rely on the cooperation of the cells being interrogated but could be influenced by presence or absence of certain receptors and adhesion molecules.

## The missing route – intracellular monitoring – what data point to its existence?

**Figure 1:**
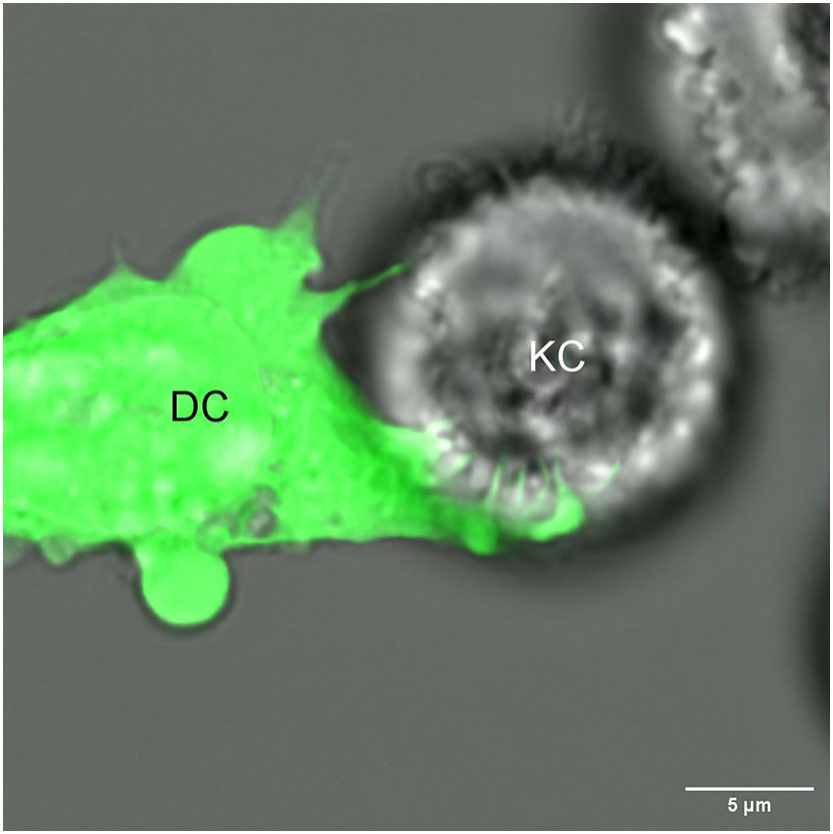
A dendritic cell (DC) extends its dendrites into a neighboring keratinocyte (KC), potentially for the purpose of intracellular monitoring.

TNTs, gap junctions and trogocytosis might contribute to intracellular monitoring by DCs. However, our recent findings that Langerhans cells (LCs) – the prototypic dendritic cell/macrophage of the epidermis (26) - unselectively and quickly acquired large quantities of keratinocyte (KC) derived mRNA and protein (27, 28), cannot be fully explained by the routes presented above. We noticed that transfer of the mRNA could be blocked by physically separating KCs from LCs with a Transwell membrane with 0.4 μm pores that allows soluble factors and vesicles, such as exosomes and cell debris up to the size of 0.4 μm to cross freely (27). LCs were also unable to acquire significant amounts of mRNA from KCs cultured on a coverslip suspended and facing the LCs and only separated from them by a few millimeters of media (unpublished observation). Altogether, these experiments eliminated the possibility that LCs were scavenging the mRNA from extracellular material, cell debris, and apoptotic bodies and implicated a contact-dependent mechanism. Time-lapse imaging shows that LCs closely interact with KCs and in matter of minutes extract mRNA from the KCs. Since this observation, we have conducted high resolution time-lapse imaging of live DC/KC co-cultures for clues on a potential mechanism. These time lapses have confirmed our previous observations, but also provided evidence DCs grabbing KCs with fang like dendritic projections (**Figure 1**). We also found that the mRNA transfer was energy dependent, required live target cells, was non-inhibitable with drugs targeting standard routes, and was not limited to LCs (unpublished observations). Thus, these data suggest the existence of a novel mechanism that allows DCs to actively and non-selectively sample other live cells’ cytosol at a very high rate.

Considerable indirect evidence also supports the existence of intracellular monitoring *in vivo*. Epidermal LCs can overcome gene deficiency if the affected protein/mRNA is expressed in the neighboring cells (29). Freshly isolated epidermal LCs contain high levels of KC-specific mRNA and proteins, such as different epidermal keratins (27). ATAC-seq confirmed that these KC-specific genes in LCs are not accessible for transcription, supporting that the keratin products were indeed acquired from KCs and not synthesized by the LCs themselves (27). The intracellular material transfer from KCs to LCs is also supported by the findings that a significant percentage of donor-derived wildtype LCs contain YFP protein and mRNA coding for YFP in irradiated hLangerin-DTA by Krt14-Cre-YFP mice (lack LCs and the KCs are labeled with YFP) reconstituted with wildtype bone marrow (27). Furthermore, YFP-positive LCs can be observed in the epidermis of the Krt14-Cre-YFP mice using two-photon microscopy, and after photobleaching, the LCs quickly recover their YFP content (unpublished observation). The presence of other cell-derived mRNA was not limited to LCs. Other tissue resident DCs also contain mRNA signature specific to their location and could be observed in human LCs (27). Altogether these data, considering our *in vitro* findings that the standard routes of antigen acquisition did not significantly contribute to the transfer of intracellular material, support the existence of intracellular monitoring *in vivo*. A phenomenon that is preserved across different species, which might play an essential role in maintaining tissue homeostasis. However, the real challenge will be to provide scientific evidence that this novel route also plays a dominant role in intracellular material acquisition *in vivo*, and to determine its immunological roles. These will require the identification of receptors and mechanistic details specific to this novel route that can then be selectively inhibited and altered.

**Figure 2:**
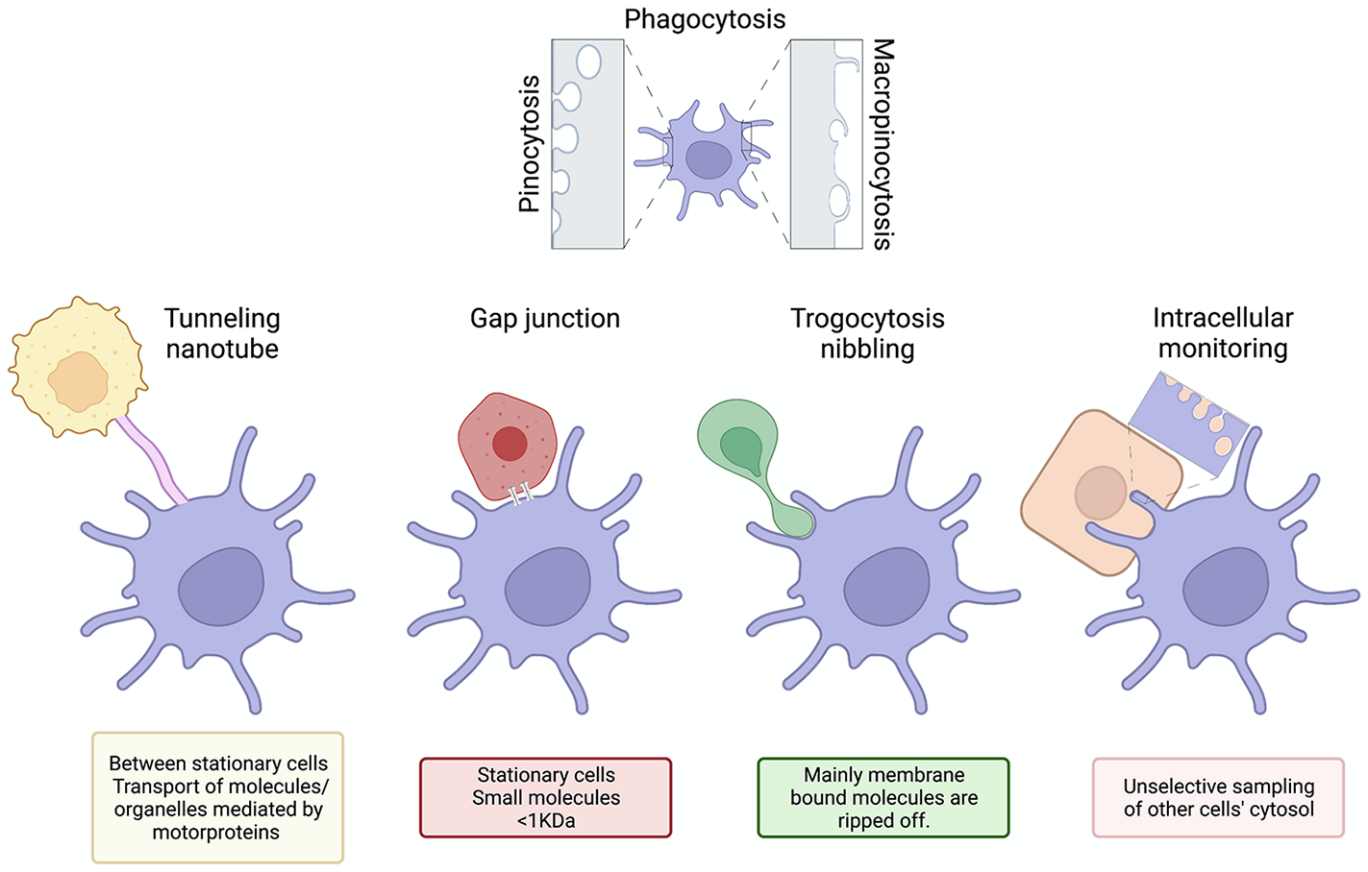
From extracellular scavenger to active intracellular monitoring. Based on the present paradigm DCs scavenge the extracellular space using processes, such as phagocytosis, pinocytosis, micropinocytosis, etc. to acquire information and antigens. Tunneling nanotubes, gap junctions and trogocytosis might also mediate the information exchange, but because of their limitations, we propose the existence of a novel mechanism by which DCs monitor and surveille other cells’ cytosol. Generated using BioRender.com

LCs constitute a unique set of cells that are macrophages by origin but with DC functions (26, 30). Whether other macrophage subsets can perform intracellular monitoring and what roles that might play in immune- and tissue homeostasis remains to be determined. Unlike circulating immune cells, some DC subsets are packed tightly in peripheral tissue, presenting unique challenges for cytoskeletal movement. With less room to maneuver and coordinate the dramatic cytoskeletal changes associated with something like phagocytosis, perhaps DC’s long branching dendrites evolved to weave through extracellular space and conduct a form of targeted, contact dependent sampling (**Figure 2**). Regardless of mechanism, we can be sure that intracellular monitoring will have a large impact on our understanding of multiple aspects of immunity. In the next section, we will explore these impacts as they pertain to infectious disease, cancer, tolerance, conditional knockout models, and the influence of the microenvironment on resident immune cells.

## What are the practical implications of intracellular monitoring?

### Intracellular pathogens

Conventionally, we know that dendritic cells become alerted to viral and intracellular bacterial infection by acquiring infected cell debris or apoptotic bodies, by becoming infected themselves, or by molecules released by the infected cells. This makes sense for lytic viruses or non-lytic viruses that happen to be DC tropic, but not for non-lytic, non-DC tropic viral infections. Such viruses would have additional time to replicate before being detected or may avoid detection altogether. This may be especially true for pathogens that transfer from cell to cell without ever entering the extracellular space like HIV (31). Intracellular monitoring would increase the likelihood of DCs encountering intracellular pathogens and save valuable time in the initiation of an adaptive response. Drugs enhancing intracellular monitoring may therefore help prevent severe disease or eliminate chronic infections. Mutations causing deficiencies in this process may also be identified as risk factors for severe infection of certain pathogens. In contrast, intimate connections between stromal cells and DCs during intracellular monitoring may be hijacked to increase the pathogen dissemination. Inhibiting intracellular monitoring may alleviate this issue.

### Cancer

It’s well established that innate immune cells are activated through ligation of pattern recognition receptors by foreign material (PAMPs), or damage associated material (DAMPs), however this requirement for receptor ligation limits the immune system to detection of specific predetermined ligands. Ideally, the immune system would not just detect whether a certain molecule is present in a cell, but also whether a cell is metabolically healthy. Considering this, the concept of a homeostasis altering molecular process, or HAMP, has been proposed, which refers to inflammasome activation by metabolic imbalances like amino acid starvation, low potassium levels, or reduced fatty acid oxidation (32). HAMP signaling is advantageous because it can detect metabolic changes characteristic of oncogenesis (33), however these signals would be difficult for immune cells to detect over the noise of competing metabolic information from healthy cells. Intracellular monitoring would facilitate the detection of HAMPs by immune cells, allowing the immune system to keep tabs on the health of a cell, not just the presence of particular molecules. Therefore, if intracellular monitoring is indeed used by DCs to detect and eliminate cancer cells, identifying targets that upregulate this process or prevent its exhaustion may be beneficial in the treatment of cancer. *Ex vivo* genetic modification of DCs to enhance intracellular monitoring followed by re-introduction into the host could be used as a novel cell therapy. Alternatively, if inhibition is possible by donor cells, identifying and blocking inhibitor molecules may diminish cancerous cells’ ability to escape detection.

Finally, cancer cells do not become cancerous by inventing new systems of cell division, apoptosis resistance, and mobility. Instead, they hijack and inappropriately utilize pathways that exist for other purposes, such as angiogenesis and epithelial to mesenchymal transition. The process of intracellular monitoring may be similarly hijacked to enable cancer cells to communicate with surrounding untransformed cells. Indeed, it is well documented that cancer cells acquire mitochondria and nutrients from surrounding cells (34). In this case, proteins facilitating intracellular monitoring could serve as novel drug targets whose blocking would cut off a tumor’s supply lines.

### Tolerance

Dissecting intracellular monitoring will be beneficial for our understanding of self-tolerance. In the steady state, DCs regularly undergo homeostatic migration from the periphery to lymph nodes while loaded with self-antigen (35–38), which they likely acquire in part through intracellular monitoring. Exactly how DCs decide when to migrate to the lymph node in the steady state is unclear, but our data suggests it may be regulated by the amount of material they have acquired. Our lab has found that LCs containing more keratinocyte derived protein are more likely to migrate in response to mechanical stimuli (27). Understanding intracellular monitoring may provide insight into the dynamics of T_reg_ induction by self-antigen laden migratory DCs in the steady state. An area that is particularly likely to involve intracellular monitoring is selection within the thymus. Transfer of antigen from medullary thymic epithelial cells to DCs is crucially important for negative selection of developing thymocytes and has been shown to be contact dependent (39). Malfunctions in this application of intracellular monitoring may therefore underlie autoimmune disorders.

Finally, rejection of tissue grafts first requires the activation of T cells specific for mismatched donor MHC molecules (40). If acquisition of MHC molecules is predominantly mediated through intracellular monitoring, inhibiting this function may improve transplant acceptance while avoiding the severe side effects of broadly acting immunosuppressants.

### Microenvironmental influence on immune cells

Tissue specific cell modification is known to occur to a significant degree in macrophages (41–43). These tissue resident cells evolve locally to fit their microenvironment, often changing nutrient usage and morphology (44). Macrophages harvested from a particular tissue used to reconstitute a different tissue will take on the characteristics of macrophages in the reconstituted tissue—often reprogramming thousands of enhancers (45). While it has not been as extensively studied, the same is likely true for tissue resident DCs. The actual mechanism of how tissue resident immune cells interact with their microenvironment and are instructed to change is not well understood. One plausible explanation would be the transfer of functional RNA through intracellular monitoring, which would enable the precise modification of tissue resident immune cells, while preventing systemic changes that may occur through the release of messengers to the extracellular environment.

### Overcome gene deficiency and/or lead to unspecific deletion

The Cre/lox system is a tool widely used by researchers to delete genes in specific cell types. Broadly, it works by utilizing a bacterial Cre recombinase to excise genomic DNA that is flanked by 34 base pair segments known as flox regions. These flox regions will be present in every cell of a mouse, however expression of the Cre recombinase is restricted to certain cell types by being placed under the control of a cell type specific promoter. If DCs continually siphon material from surrounding cells, it is likely that they will acquire Cre recombinase even if they do not express it themselves. This would result in off target effects. Indeed, we found that KC-derived Cre led to genetic recombination in LCs (27).

Alternatively, Cre/lox gene deletions targeted to DCs may be overcome if DCs siphon the deleted protein from surrounding cells. This may explain why some Cre/lox induced gene deletions do not result in a detectable phenotype. Our lab has observed that LCs in the epidermis overcome gene deletion of Cx43 and MyD88, which are present and can be siphoned from surrounding keratinocytes, but not MHC-II (29), which is absent in keratinocytes.

## Conclusion

Dendritic cells are the crucial link between innate and adaptive immunity. To prime T cells, DCs must first acquire antigen and be alerted to danger. Traditionally, DCs do this through pinocytosis, phagocytosis, macropinocytosis, or by direct infection. However, our lab has identified the transfer of significant quantities of mRNA and protein from stromal cells to DCs in the steady state that cannot be accounted for by these means. While tunneling nanotubes and trogocytosis could contribute to this type of material transfer, we propose that an additional, contact dependent mechanism of transfer is used by DCs to routinely monitor the cytosol of surrounding cells in a process termed intracellular monitoring. Such a process likely evolved to provide dendritic cells—the sentinels of the immune system—access to substantially more information about their surroundings, enhancing their capacity to detect infections and cancer, and adapt to their microenvironment. Considering its advantages and mechanistic feasibility, we believe intracellular monitoring will be a fruitful area of research in the next decade with many potential therapeutic applications.

## Data availability statement

The original contributions presented in the study are included in the article/supplementary material. Further inquiries can be directed to the corresponding author.

## Author contributions

CH wrote the draft. LH edited the draft. BI conceptualized the manuscript, edited the draft, and proposed the terminology of intracellular monitoring/surveillance. All authors contributed to the article and approved the submitted version.

## Funding

BZI is supported by the National Institute of Allergy and Infectious Diseases R01AI146420 and R01AI146101, and institutional start-up funds.

## Conflict of interest

The authors declare that the research was conducted in the absence of any commercial or financial relationships that could be construed as a potential conflict of interest.

## Publisher’s note

All claims expressed in this article are solely those of the authors and do not necessarily represent those of their affiliated organizations, or those of the publisher, the editors and the reviewers. Any product that may be evaluated in this article, or claim that may be made by its manufacturer, is not guaranteed or endorsed by the publisher.
